# Group 1 metabotropic glutamate receptors 1 and 5 form a protein complex in mouse hippocampus and cortex

**DOI:** 10.1002/pmic.201500400

**Published:** 2016-09-12

**Authors:** Nikhil J. Pandya, Remco V. Klaassen, Roel C. van der Schors, Johan A. Slotman, Adriaan Houtsmuller, August B. Smit, Ka Wan Li

**Affiliations:** ^1^ Department of Molecular and Cellular NeurobiologyCenter for Neurogenomics and Cognitive ResearchNeuroscience Campus AmsterdamVU UniversityAmsterdamthe Netherlands; ^2^ Optical Imaging CenterErasmus Medical CenterRotterdamthe Netherlands

**Keywords:** Animal Proteomics, Brain, G‐protein coupled receptor, Immunoprecipitation, Mass spectrometry

## Abstract

The group 1 metabotropic glutamate receptors 1 and 5 (mGluR1/5) have been implicated in mechanisms of synaptic plasticity and may serve as potential therapeutic targets in autism spectrum disorders. The interactome of group 1 mGluRs has remained largely unresolved. Using a knockout‐controlled interaction proteomics strategy we examined the mGluR5 protein complex in two brain regions, hippocampus and cortex, and identified mGluR1 as its major interactor in addition to the well described Homer proteins. We confirmed the presence of mGluR1/5 complex by (i) reverse immunoprecipitation using an mGluR1 antibody to pulldown mGluR5 from hippocampal tissue, (ii) coexpression in HEK293 cells followed by coimmunoprecipitation to reveal the direct interaction of mGluR1 and 5, and (iii) superresolution microscopy imaging of hippocampal primary neurons to show colocalization of the mGluR1/5 in the synapse.

**Abbreviations**
FASP: filter‐aided sample preparation
IP: immunoprecipitation
mGluR1/5: metabotropic glutamate receptor 1 and 5
SIM: structured illumination superresolution microscopy

## 1 Introduction

Group1 metabotropic glutamate receptors are G‐protein coupled receptors comprising the closely related mGluR1 (GRM1) and 5 (GRM5), where mGluR1 exists in two isoforms differing only at the C‐termini due to alternative splicing of the gene 1. Metabotropic GluR5 is primarily localized at the postsynapse, where it regulates short‐ and long‐term synaptic plasticity, in particular long‐term depression 2. Activation of metabotropic glutamate receptor 1 and 5 (mGluR1/5) leads via Gq/G_11_ proteins to the initiation of phospholipase‐C_β_ thereby eliciting IP3 and diacylglycerol signaling 3. In addition, mGluR1/5 activation can lead to the activation of MAPK/ERK and MTOR/p70 S6 kinase, which are involved in synaptic plasticity 3, 4. Nonsynaptic mGluR5 also exists, and was recently shown to activate different signaling systems than synaptic mGluRs 5. Negative or positive allosteric modulators of mGluR5 have therapeutic potential for a number of brain disorders including fragile X mental retardation and schizophrenia 6. Accordingly, mGluR5 is considered a promising drug target aimed at alleviating various neurological and psychiatrically disorders by pharmacological intervention of the receptor activity 3, 7, 8.

**Significance of the study**

Interaction proteomics has the potential to identify protein interactions that take part in the formation of multicomponent complexes. For the first time, we used a knockout‐controlled interaction proteomics strategy to investigate the mGluR5 interactome directly from the hippocampus and the cortex and found that constituents of the mGluR5 complex are similar between the cortex and the hippocampus. We show that a population of mGluR5 forms a stable complex with mGluR1 in both brain regions. These findings have implications in the design and study of allosteric modulators of heteromeric receptor complexes and the understanding of the role of these receptors and their modulators in vivo. Furthermore, the immunoprecipitation protocol combined with a filter‐aided sample preparation protocol paves the way for large‐scale high‐content interaction proteomics studies for other protein receptor complexes in the brain.

Besides G‐proteins, also phosphatases (PP1γ1) 9, kinases (PKC) 10, scaffolding proteins (NHERF‐2, Tamalin/GRASP) 11, 12, and ion channels (Grid2) 13, have been reported as being part of the group1 mGluR signaling complexes, and driving diverse cellular processes, such as, subcellular localization and Ca^2+^ responses presumably in a brain region specific manner. mGluR5 is abundantly present in the hippocampus and mGluR1 has a higher expression in cerebellum 14. As functions of mGluRs critically depend on their interacting proteins, it is important to elucidate the constituents of these protein complexes. Attempts have been made to elucidate the mGluR1/5 interactome, but with variable success due to the use of only an in vitro model and/or ambiguity of some interactors 15, 16.

In the present study, we used a knockout‐controlled interaction proteomics analysis to examine the interactome of mGluR5 in hippocampus and cortex. Apart from the known interacting proteins, Homer1‐3, two mGluR1 isoforms, mGluR1a and 1b, form a complex with mGluR5. We confirmed the presence of the mGluR5‐1a/b complex in hippocampus by reverse immunoprecipitation (IP), revealed the direct interaction of mGluR1/5 by coimmunoprecipitation from HEK293 cells coexpressing both receptors, and demonstrated the colocalization of mGluR1/5 at the postsynapse by structured illumination superresolution microscopy (SIM) imaging.

## 2 Materials and methods

### 2.1 Sample preparation and IP

Whole hippocampus and cortex were dissected from mouse brains, and stored at −80°C until used. The brain issue was homogenized in 1% freshly prepared *n*‐dodecyl β‐d‐maltoside containing 25 mM HEPES (pH 7.4) and 150 mM NaCl and a protease inhibitor cocktail (Roche), in a PotterS homogenizer with 12 strokes at 900 rpm and incubated for 1 h at 4°C. After centrifugation at 20 000 × *g* for 20 min the supernatant was collected and centrifuged again at 20 000 × *g* for 20 min. The final supernatant was used for IP, with the equivalent of one hippocampus and 0.3 cortex per IP experiment. Ten micrograms of antibody was added to each sample, and incubated overnight at 4°C with rotation. Sixty microliters Protein A/G plus agarose bead (Santa Cruz) was used per IP to capture the antibody. After washing, the agarose beads containing the protein complex were subjected to filter‐aided sample preparation (FASP) treatment (as described below).

The anti‐mGluR5 polyclonal antibody was obtained from Genscript (A01493); the anti‐mGluR1a monoclonal antibody was obtained from BD Biosciences (clone G209‐488). These antibodies were also used for immunoblot analysis and immunostaining of primary neurons.

### 2.2 FASP in‐solution digestion of proteins

FASP was used with small modifications 17. In short, agarose beads from an IP experiment were gently vortexed in 75 μL 2% SDS, 1 μL 50 mM Tris (2‐carboxyethyl)phosphine at 55°C for 1 h. The reduced cysteines were blocked by incubation with 0.5 μL 200 mM methyl methanethiosulfonate for 15 min at RT. The sample was centrifuged at 16 000 × *g* for 20 min. The supernatant was mixed with 200 μL 8 M Urea in Tris pH 8.8, transferred to Microcon‐30 (Millipore, Lot R4NA17256), and centrifuged at 14 000 × *g* for 12 min at RT. The addition of 200 μL 8 M urea to the filter and centrifugation were repeated three times. To remove urea, four serial washes were performed with the addition of 200 μL 50 mM NH_4_HCO_3_ for each wash followed by centrifugation as stated above. Samples were digested with 0.7 μg Trypsin/Lys‐C Mix (MS grade from Promega) in 100 μL 50 mM NH_4_HCO_3_ overnight in a humidified chamber at 37°C. Hundred microliters of 0.1% acetic acid was added to the filter, and centrifuged. The tryptic peptides were collected, dried in a speedvac, and stored at −20°C until LC–MS analysis.

### 2.3 Preparation of P1, P2, and microsome fractions

P1, P2, and Microsome fractions were prepared as in 18. Briefly, hippocampi were homogenized in ice‐cold homogenization buffer (0.32 M sucrose, 5 mM HEPES (pH 7.4)) containing protease inhibitor (Roche). Pellet 1 (P1) was obtained after spinning at 1000 × *g* at 4°C for 10 min. The supernatant was spun at 20 000 *x g* at 4°C to obtain Pellet 2 (P2). The supernatant from P2 was further centrifuged at 100 000 × *g* for 2 h to obtain a microsome fraction.

### 2.4 In‐gel separation digestion of proteins

IPs were performed as before on an equal amount (2 mg) of P2, microsome, and P1 fractions from hippocampus. SDS‐PAGE electrophoresis and in‐gel digestion were performed as described 19.

### 2.5 LC–MS/MS acquisition and data analysis

Peptides were analyzed by two types of nano‐LC MS systems, namely LTQ‐Orbitrap Discovery (Thermo Scientific) and TripleTOF 5600+ (Sciex) MS. For the analysis using TripleTOF 5600+ MS, it was coupled with an Ultimate 3000 LC system (Dionex, Thermo Scientific). Peptides were trapped on a 5 mm Pepmap 100 C18 column (300 μm id, 5μm particle size, Dionex) and fractionated on a 200 mm Alltima C18 column (100 μm id, 3 μm particle size). ACN concentration in the mobile phase in 0.1% formic acid was increased from 5 to 18% in 88 min, to 25% at 98 min, 40% at 108 min, and to 90% at 110 min, at a flow rate of 400 nL/min. Peptides were electrosprayed into the mass spectrometer using an ion spray voltage of 2.5 kV, curtain gas at 35 p.s.i., nebulizer gas at 15 p.s.i., and an interface heater temperature of 150°C. The MS survey scan range was *m/z* 350–1250 acquired for 250 ms. The top 20 precursor ions were selected for 85 ms per MS/MS acquisition, with a threshold of 90 counts. Dynamic exclusion was 16 s. Rolling CID function was activated, with an energy spread of 15 eV.

For the analysis using the LTQ‐Orbitrap MS, peptides were loaded onto a nano‐LC Ultra system (Eksigent) with the trapping and separation columns as described for 5600+ MS analysis. ACN concentration in 0.1% acetic acid was linearly increased from 5 to 40% in 80 min and to 90% in 10 min, and electro‐sprayed into the LTQ‐Orbitrap MS using an ion spray voltage of 1.4 kV and heater temperature of 200°C. LTQ‐Orbitrap was operated in the range of *m/z* 350–2000 at a FWHM resolution of 30 000 after accumulation to 500 000 in the LTQ with one microscan. The five most abundant precursor ions were selected for fragmentation by CID with an isolation width of 2 Da.

All raw MS data were analyzed by MaxQuant software (version 1.5.2.8) with search engine Andromeda. The Mouse database used was UniProt_2015‐02. The fixed modification was MMTS (for FASP) and propioamide (for in‐gel digestion samples). Match between runs with match time window of 0.7 min and alignment time window of 5 min were used for all analyses. For other parameters the default settings were used.

### 2.6 Expression plasmids

Mouse mGluR1, transcript variant 1 (NM_016976.3), was Gateway‐cloned into the pReceiver‐Lv186 vector, yielding mGluR1‐pReceiver‐Lv186 (including a C‐terminal 3xHA‐tag) (GeneCopoeia; catalog number EX‐Mm02865‐Lv186). Mouse mGluR5, transcript variant a (NM_001081414), was cloned into the pcDNA3.1(+) vector, yielding mGluR5‐pcDNA3.1 (Genscript).

### 2.7 HEK293 cell culture and transfection

HEK293 cells (ATCC) were cultured in DMEM medium (Gibco, Life Technologies), 10% FBS (Invitrogen), and 1% penicillin‐streptomycin (Gibco, Life Technologies) in 10 cm dishes. Cells were 60–70% confluent at the time of transfection and of passage number 14. Medium was refreshed 2–3 h prior to transfection. HEK293 cells were transfected with plasmid DNA (5 μg) using polyethylenimine (25 kDa linear, Polysciences) and incubated for 48 h after transfection.

### 2.8 Coprecipitation from HEK293 cells

All steps were performed at 4°C, with the exception of elution (room temperature). For protein extraction, HEK293 cells were washed with PBS, resuspended in freshly prepared lysis‐buffer (1% DDM, 25 mM HEPES (pH 7.4), 150 mM NaCl, and EDTA‐free Complete protease inhibitor (Roche)), and incubated for 1 h with gentle end‐over‐end mixing. The supernatant was cleared of nonsoluble debris by two consecutive centrifugation steps at 20 000 × *g* for 15 min. Anti‐mGluR1 antibody (4 ug) or anti‐mGluR5 antibody (4 ug) was added to the supernatant, incubated O/N, and immobilized to Protein A/G agarose beads (Santa Cruz). The agarose beads were washed four times with wash buffer (0.1% DDM, 25 mM HEPES, and 150 mM NaCl), and the bound proteins were eluted by incubation with 2× Laemmli sample buffer. Input samples were prepared from the supernatant fraction by addition of Laemmli sample buffer to a 2× final concentration.

### 2.9 SDS‐PAGE and immunoblot analysis

Protein samples were heated to 55°C for 45 min prior to loading onto a 4–15% Criterion TGX Stain‐Free Precast gel (Bio‐Rad), with the input samples representing 3% of the lysate used for IP. The gel‐separated proteins were imaged with the Gel‐Doc EZ system (Bio‐Rad), directly transferred onto Immun‐Blot PVDF membrane (Bio‐Rad) and probed O/N at 4°C with mGluR1 antibody (1:1000) or mGluR5 antibody (1:1000). Chemiluminescence scans were acquired with the Odyssey Fc system (Li‐Cor), and analyzed using Image Studio Lite 5.2.5 software (Li‐Cor).

### 2.10 Protein molecular weight prediction

The predicted molecular weight was determined using the Expasy online tool (http://web.expasy.org/compute_pi/). The predicted molecular weight for mGluR1 as expressed by the mGluR1‐pReceiver‐Lv186 plasmid (including a C‐terminal 3xHA‐tag) was 137.6 kDa. The predicted weight for mGluR5 was 128.3 kDa.

### 2.11 Immunostaining of primary neurons

Primary hippocampal neurons were obtained from E18 rat pups. Briefly, 18 000 cells were grown in neurobasal medium supplemented with B27 on poly d‐Lysine coated coverslips. The cells were used for staining at DIV 14–16. The coverslips were fixed with ice‐cold methanol for 10 min, followed by three washes in ddH_2_O and PBS. The neurons were then blocked and permeablized with blocking buffer (5% FCS, 0.1% Triton X‐100, and 0.1% glycine in phosphate buffer saline) for 1 h. Next, the neurons were incubated with anti‐mGluR1 or anti‐mGluR5 antibodies, and anti‐Homer1 (cat. no. 160 004**,** Synaptic systems), diluted in blocking buffer overnight at 4°C. After washing three times in PBS, the cells were incubated with an alexa conjugated secondary antibody for 1 h at room temperature (anti‐rabbit Alexa 488 (1 in 1000), anti‐mouse Alexa 568 (1 in 1000), anti‐Guinea pig Alexa 647 (1 in 1000) (Molecular Probes) and subsequently washed and fixed on glass slides (Superfrost Plus, Thermo) using Moviol. Images were taken using a Zeiss Elyra PS1 SIM microscope with 63× oil immersion lens (N.A. 1.4) and reconstructed images were analyzed using ImageJ.

## 3 Results and discussion

In the present study, we employed interaction proteomics to reveal the mGluR5 interactome. Generally, antibody‐based interaction proteomics experiments are noisy, i.e. they are populated by consistently present background protein contaminants as well as by a large number of proteins that appear sporadically in the individual IPs. Here, we used an mGluR5 knockout mouse as a negative control to remove false positives, which in addition should address the problem of (potential) cross‐reactivity of the antibody. We carried out multiple IP replicates to filter out the sporadically occurring proteins. As we focused on stable mGluR complexes, the protein constituents of the complexes should be present in most, if not all, IPs. We took a stringent filter to select interacting proteins, which must be present in ≥3 of 4 IPs from WT samples and have an enrichment factor of ≥10‐fold. From the approximately 300 identified proteins in the mGluR5 IPs from extracts of hippocampus and cortex (excluding external contaminants, such as keratins, antibodies, trypsin, and bovine proteins), 14 proteins pass this filter (Table 1). The bait protein mGluR5 has the highest intensity, followed by Homer1 and mGluR1. mGluR1 is present at a much lower amount than mGluR5, suggesting that a fraction of mGluR5 protein complexes contain mGluR1. Proteins of lower intensity in the IP table mostly represent high abundant proteins in the original extract materials, notably mitochondrial proteins and proteins involved in energy metabolism, both of which are often considered as the major sources of contaminants in typical IP experiments. In case a protein passed the filter in one brain region (for example Rab3gap1 and Atp1a2) but was detected in KO samples from another brain region at a level of those of WT, they may present false positives and therefore should be considered with caution (see also Table 2). The present, as well as our previous studies, identified 200–300 proteins from a single IP. To reveal true positives, stringent filter(s) were applied, together with the use of negative control(s), such as the appropriate knockout mouse samples or alternatively the antigen peptide blocking approach. Furthermore, reverse IP was used to confirm the interaction 19. In this context, a recent interaction proteomics study highlighted a similar example in which 495 proteins were identified from an IP, which eventually was filtered to 16 interactors 20. Recently, using the same strategy we examined the interactome of an adhesion molecule, Caspr2 (CNNTP2), and revealed tens of proteins as genuine interactors 21. Here, we revealed a simple composition of mGluR5 interactome with Homer1, 2, and 3 and mGluR1a/b as main interactors. This interactome is simple in contrast to, for instance, the complex interactome of the ionotropic AMPA‐type glutamate receptors, which is reported to contain up to 30 associated proteins 22, 23, 24. A G‐protein coupled receptor, such as mGluR5, may exhibit many transient interactions during its activation as exemplified by the interaction with G‐protein subunits. These short‐lived interactions of presumably relatively low affinity may not be recovered in IP. This might explain that only a small but stable mGluR5 interactome is revealed under the present experimental conditions.

**Table 1:** Intensities of mGluR5 interactors immunoprecipitated from cortex and hippocampus

Gene Names	CORTEX								HIPPOCAMPUS							
	KO‐1	KO‐2	KO‐3	KO‐4	WT‐1	WT‐2	WT‐3	WT‐4	KO‐1	KO‐2	KO‐3	KO‐4	WT‐1	WT‐2	WT‐3	WT‐4
Grm5	4 × 10^6^	1 × 10^6^	—	—	2 × 10^9^	2 × 10^9^	1 × 10^9^	2 × 10^9^	—	—	—	1 × 10^6^	1 × 10^9^	2 × 10^9^	1 × 10^9^	1 × 10^9^
Homer1	—	—	—	—	3 × 10^8^	2 × 10^8^	1 × 10^8^	1 × 10^8^	—	—	—	—	5 × 10^7^	1 × 10^8^	1 × 10^8^	1 × 10^8^
Grm1 (isoform b)	—	—	—	—	2 × 10^7^	2 × 10^7^	2 × 10^7^	3 × 10^7^	—	—	—	—	1 × 10^7^	4 × 10^7^	2 × 10^7^	1 × 10^7^
Homer3	—	—	—	—	—	—	—	—	—	—	—	—	4 × 10^6^	2 × 10^6^	1 × 10^7^	1 × 10^7^
Homer2	—	—	—	—	3 × 10^6^	7 × 10^6^	6 × 10^6^	3 × 10^6^	—	—	—	—	6 × 10^6^	3 × 10^6^	1 × 10^7^	8 × 10^6^
Grm1 (isoform a)	—	—	—	—	4 × 10^6^	7 × 10^6^	4 × 10^6^	3 × 10^6^	—	—	—	—	2 × 10^6^	1 × 10^7^	4 × 10^6^	4 × 10^6^
Grm3	—	—	—	—	1 × 10^6^	—	1 × 10^6^	1 × 10^6^	—	—	—	—	—	—	—	5 × 10^5^
Clu	—	—	—	—	3 × 10^6^	2 × 10^6^	1 × 10^6^	3 × 10^6^	—	—	—	—	2 × 10^6^	2 × 10^6^	2 × 10^6^	—
Rab3gap1	—	—	—	—	6 × 10^8^	6 × 10^8^	8 × 10^8^	9 × 10^8^	—	8 × 10^8^	8 × 10^8^	—	—	7 × 10^8^	—	—
Atp1a2	—	—	7 × 10^5^	1 × 10^6^	—	—	8 × 10^5^	1 × 10^6^	—	—	—	—	2 × 10^6^	—	3 × 10^6^	2 × 10^6^
*Hbbt1; Hbb‐bs*	—	—	—	—	6 × 10^6^	3 × 10^6^	7 × 10^6^	6 × 10^6^	5 × 10^6^	—	1 × 10^6^	—	5 × 10^7^	4 × 10^6^	1 × 10^7^	2 × 10^7^
*Slc3a2*	—	—	–	—	—	—	7 × 10^5^	6 × 10^5^	—	—	—	–	2 × 10^6^	—	1 × 10^6^	3 × 10^6^
*Glud1*	—	—	—	—	—	—	—	—	—	—	—	—	2 × 10^6^	—	2 × 10^6^	2 × 10^6^
*Atp6v1e1*	—	—	—	—	—	—	—	—	—	—	—	—	2 × 10^6^	—	2 × 10^6^	1 × 10^6^

Maxquant MS1 peak intensities for the proteins quantified for unique peptides obtained from the mGluR5 IPs are listed here. Proteins are at least tenfold enriched in WT, and observed at least three of four times in WT. Gene names in italic are present in empty beads experiment in Table 2, and most likely represent false positives.

KO: mGluR5 IP from knockout mice; WT: mGluR5 IP from wild‐type mice.

**Table 2:** Intensities of mGluR5 interactors immunoprecipitated from hippocampal subfractions

Gene Names	Microsome			P1			P2		
	EB	IP_1	IP_2	EB	IP_1	IP_2	EB	IP_1	IP_2
Grm5	—	4 × 10^8^	3 × 10^8^	—	3 × 10^8^	3 × 10^8^	—	2 × 10^8^	2 × 10^8^
Grm1 (isoform a)	—	490 210	444 620	—	330 400	391 060	—	262 450	162 310
Grm1 (isoform b)	—	—	852 620	—	825 770	828 400	—	300 820	694 750
Homer1	—	7 × 10^6^	1 × 10^7^	—	8 × 10^6^	1 × 10^7^	—	8 × 10^6^	7 × 10^6^
Homer3	—	526 800	606 300	—	2 × 10^6^	2 × 10^6^	—	2 × 10^6^	2 × 10^6^
*Atp6v1e1*	425 540	693 690	482 760	247 240	—	—	297 740	461 910	366 990
*Glud1*	305 810	349 040	—	—	—	—	623 670	—	371 350
*Hbbt1; Hbb‐bs*	2 × 10^6^	2 × 10^6^	589 360	2 × 10^6^	2 × 10^6^	2 × 10^6^	3 × 10^6^	1 × 10^6^	—
*Slc3a2*	—	—	—	174 410	—	—	157 460	—	—

Maxquant MS1 peak intensities for the proteins quantified for unique peptides obtained from the mGluR5 IPs are listed here. Proteins in italic are the putative mGluR5 interactors shown in Table 1. They are present in the empty bead controls and most likely represent false positives.

EB: Empty bead control; P1: Pellet 1; P2: Pellet 2.

Homer 1, 2, and 3 were previously described as mGluR5 interactors, and indeed were recovered from our IPs at high scores (>10^8^). Homer 1, 2, and 3 of the homer family, exhibited a distinct brain region‐specific mGluR5 interaction pattern, which closely reflects their differential gene expression patterns in these brain regions. In particular, Homer 1 and 2 are abundantly expressed in cortex and hippocampus, whereas Homer 3 is higher expressed in hippocampus but low in cortex. It is of notice that differential spatial expression occurs even within a brain region; in hippocampus Homer 1 and 2 show major expression in the CA1 region, whereas Homer 3 has highest expression in CA3 (see Allen Brain Atlas Mouse Brain ISH for Homer 1, 2, 3). Future studies will be needed to resolve these protein complexes in order to reveal their similarity/differences.

Although mGluR5 is abundantly present in the postsynapse, it is also found extrasynaptically in endoplasmic reticulum as well as in astrocytes 25. The activation of intracellular dendritic pool of mGluR5 can mediate Ca^2+^ responses in dendrites and are sufficient for mediating LTD in hippocampal neurons 26. To examine if there is difference of mGluR5 protein complexes present in distinct subcellular structures, we performed IP on biochemically fractionated hippocampal subfractions, namely the P1 fraction enriched in cell bodies, the synapse‐enriched P2 fraction, and the microsome fraction that is enriched for endoplasmic reticulum, Golgi, and vesicles 27. The mGluR5 complex composition was similar across different fractions (Table 2). It will be interesting to explore the subcellular localization of mGluR5–Homer complex, which might be present outside the postsynapse and thereby confirm the biochemical fractionation data. Specific interactions may underlie distinct functions, such as receptor trafficking or receptor‐regulating roles. The role of Homer1 in stabilization of mGluR5 scaffolds has been well documented, however the functional implications of mGluR1‐mGluR5 complexes have not been studied in much detail. Several potential interacting proteins were found (Table 1). Glud1, Atp6ve1, hemoglobin, and Scl3a2 were found in empty bead controls in this experiment (Table 2), and/or not consistently observed in mGluR5 IPs and therefore likely are false positives. The proteomics Maxquant output files for all the IP experiments are shown in Supporting Information Tables 1, 2, 3.

**Table 3:** mGluR1a IP from hippocampus confirms the mGluR1‐5 interaction

Gene names	HIPPOCAMPUS			
	Ig‐1	Ig‐2	BD‐1	BD‐2
Grm1 (isoform a)	—	—	29 026	52 842
Grm1 (isoform b)	—	—	4388.1	3858.6
Grm5	—	—	2044.3	10 978
Homer3	—	—	10 924	6548.8

Maxquant MS1 peak intensities for the proteins quantified for unique peptides obtained from the mGluR1 IPs are listed here.

BD: IP performed with mGluR1a antibody from BD Biosciences; Ig: IP performed with non‐mGluR1 antibody as negative control.

In this study, we identified two isoforms of mGluR1 in the mGluR5 IPs from cortex and hippocampus extracts. mGluR1a and 1b are generated by alternative splicing and differ at the C‐termini; mGluR1b differs from 1a at residue number 887–906 with the substitution of peptide sequence NSNGKSVSWSEPGGRQAPKG of mGluR1a to KKRQPEFSPSSQCPSAHVQL, and the remaining amino acid sequence (residues 907–1199) in mGluR1a is missing. As a consequence there is only one unique (tryptic) peptide RQPEFSPSSQCPSAHVQL (Supporting Information Fig. 1) for 1b isoform, compared to multiple unique peptides for 1a isoform.

To confirm the mGluR1‐5 interaction specific protein complex, we used a monoclonal anti‐mGluR1a antibody for IPs. Several proteins were present in both mGluR1a IPs with >10‐fold enrichment compared to the negative controls (Table 3). In hippocampus, the mGluR1a complex contained mGluR1b, mGluR5, and Homer3. These observations are in general agreement with the results of mGluR5 IPs. Homer 1 and 2, that were abundantly present in hippocampal mGluR5 IPs, were not detected in mGluR1a IP in the hippocampus. Together, this indicates that (i) mGluR1‐specific protein complex harbors primarily Homer 3, and (2) heterodimerization of mGluR1a‐b and mGluR5 can exist in neurons.

**Figure 1:**
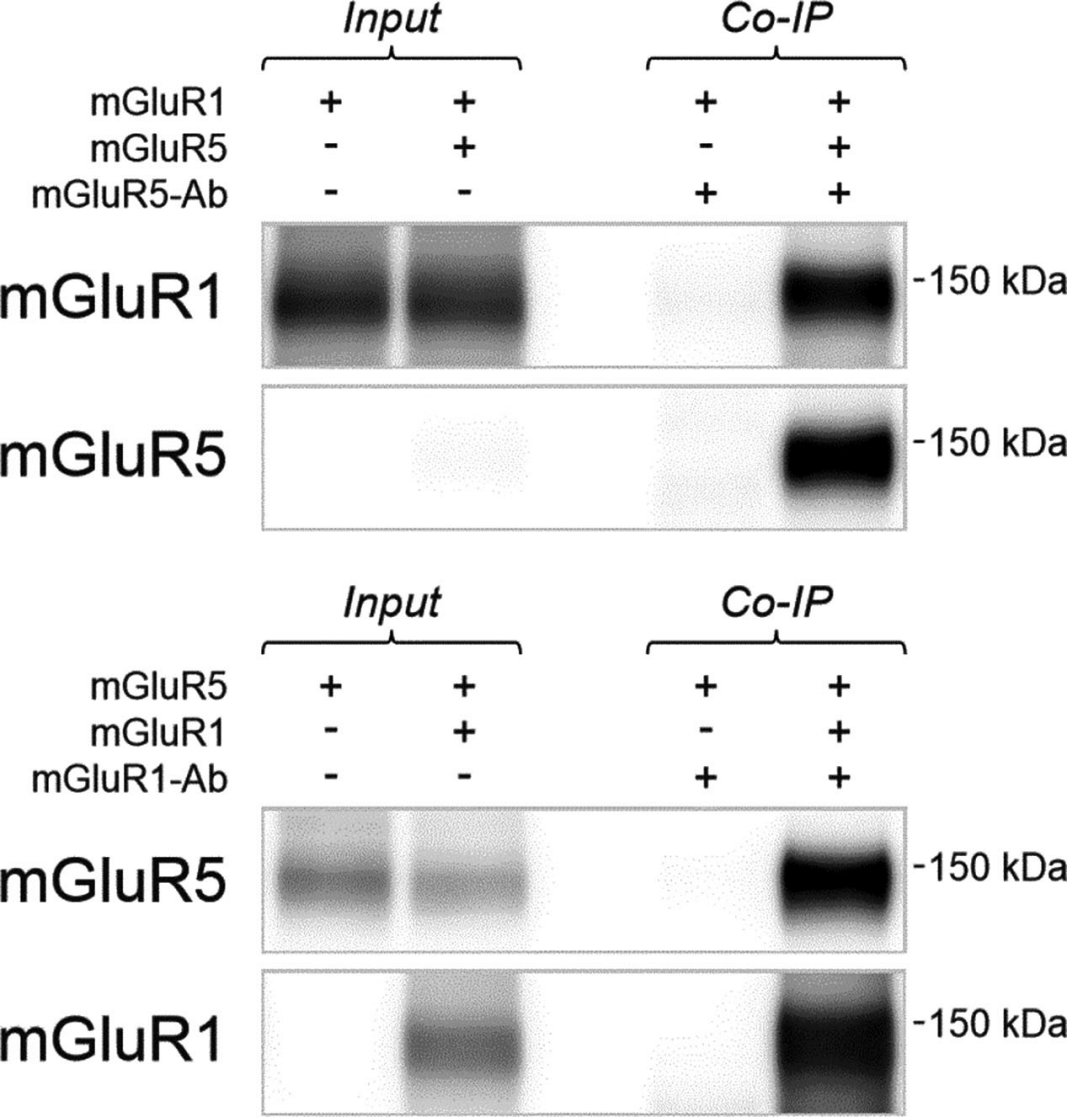
mGluR1 coimmunoprecipitates with mGluR5 in HEK293 cells. Upper panel: mGluR1 was expressed in the presence or absence of mGluR5 in HEK293 cells. Immunoprecipitation (IP) using an mGluR5‐specific antibody, and subsequent immunoblot analysis, revealed the specific coprecipitation of mGluR1 with mGluR5. Lower panel: Reverse conditions; expression of mGluR5 in the presence or absence of mGluR1, followed by mGluR1 IP, confirmed the specific coprecipitation of mGluR5 with mGluR1.

To examine whether mGluR1‐5 interact directly, we performed co‐IP experiments with expression of mGluR1 and/or 5 in HEK293 cells (Fig. 1). We confirmed the direct interaction of mGluR1/5 in the double expression of mGluR1 and 5, in which the IP of mGluR5 precipitated mGluR1 and the IP of mGluR1 precipitated mGluR5. We further confirmed the antibodies specificity; anti‐mGluR1 antibody did not stain mGluR5 and did not precipitate mGluR5, and vice versa anti‐mGluR5 antibody did not stain mGluR1 and did not precipitate mGluR1.

**Figure 2:**
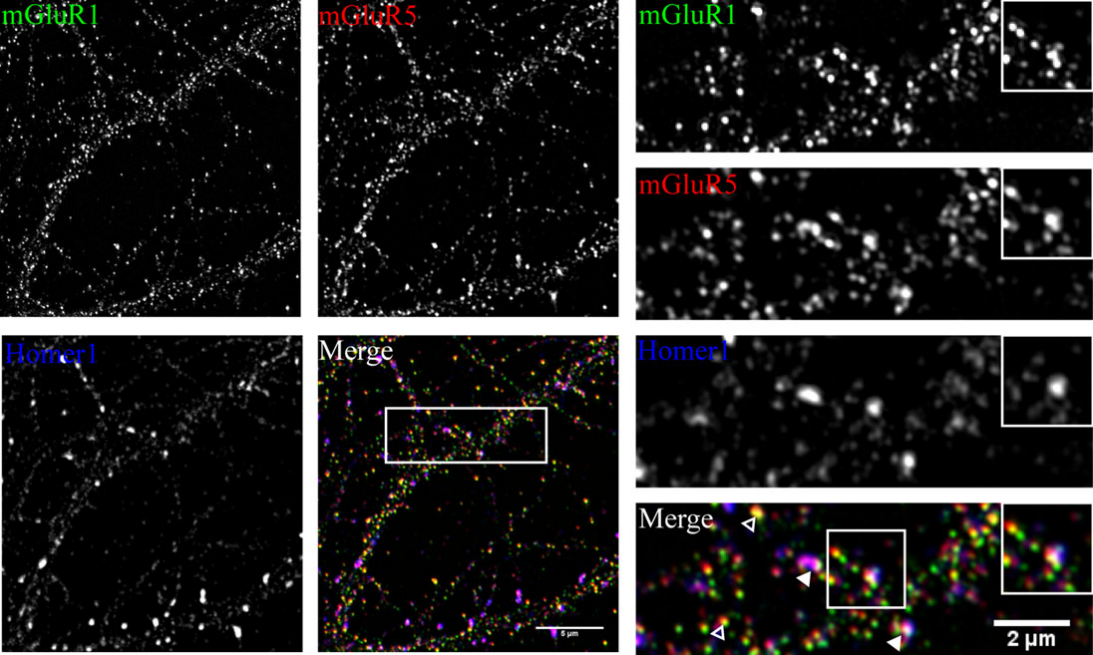
Superresolution imaging validation of mGluR1‐5 colocalization. SIM imaging in cultured hippocampal neurons at DIV 19 showing an overview or zoom‐ins of dendrites (right) or spines (right, inset). mGluR1 (green), mGluR5 (red) and, Homer1 (blue, synaptic marker) are shown. Merge shows color‐overlay images of the three channels. Scale bars are indicated. Merge panel: open arrows indicate non synaptic overlap between mGluR1 and 5, closed arrows indicate synapses positive for mGluR1 and 5. Note that extrasynaptically a large part of the mGluR1 is not colocalized with mGluR5.

If mGluR1 and 5 form a heteromeric complex, they should colocalize in the cell. We performed coimmunostaining of mGluR1 and 5 on hippocampal primary neurons and visualized these proteins with SIM imaging. The mGluR1, 5 colocalization was observed in synapses as indicated by their colocalization with the postsynapse marker protein Homer, and was also detected outside synapses (Fig. 2). There was also mGluR5 that did not colocalize with mGluR1. Together, these imaging data are supportive to our interaction proteomics data that mGluR1/5 protein complex exists, and that this complex constitutes a subpopulation of synaptic mGluR5 (Table 1).

## 4 Concluding remarks

Although we demonstrated for the first time the presence of mGluR1‐5 protein complex in the brain, interaction of mGluR1 and 5 has been implicated previously. Chemical LTD induced by selective mGluR5 agonist DHPG was abolished in mGluR5 knockout mouse 28. However, in hippocampus the complete blockade of chemical LTD required the combination of both mGluR1 and five receptor antagonists 29, suggesting heterodimerization of the two receptors. Time‐resolved fluorescence resonance energy transfer with the cell‐surface labeling of tagged rat mGluR subunits expressed in a mammalian cell line revealed that group II and III mGluR subunits can produce intergroup heteromeric receptors that are functional, whereas group I mGluR subunits, i.e. mGluR1 and 5, can interact but do not associate with groups II and III mGluR subunits 30. Recently, mGluR2/4 heterodimers have been found in the striatum at the corticostriatal synapse, and exhibit a distinct pharmacological profile 31. Functional interdependence of mGluR1 homodimer and mGluR5 homodimer has been indicated in sympathetic rat neurons coexpressing both receptors 32. This study also provides evidence of two pools of mGluR5 in striatal neurons, one of them probably interacting with mGluR1. The discovery of mGluR heteromerization represents a new avenue for the study of mGluR pharmacology and neurobiology, of which the in vivo consequences remain to be investigated.

*The authors have declared no conflict of interest*.

## Supporting information

As a service to our authors and readers, this journal provides supporting information supplied by the authors. Such materials are peer reviewed and may be re‐organized for online delivery, but are not copy‐edited or typeset. Technical support issues arising from supporting information (other than missing files) should be addressed to the authors.

Suppl. Figure S1

Suppl. Table S1

Suppl. Table S2

Suppl. Table S3
