# Supplementary figures and images for: Group 1 metabotropic glutamate receptors 1 and 5 form a protein complex in mouse hippocampus and cortex
Source: Proteomics. 2016 Sep 12;16(20):2698–705. doi: 10.1002/pmic.201500400 (PMC5129514; doi:10.1002/pmic.201500400)

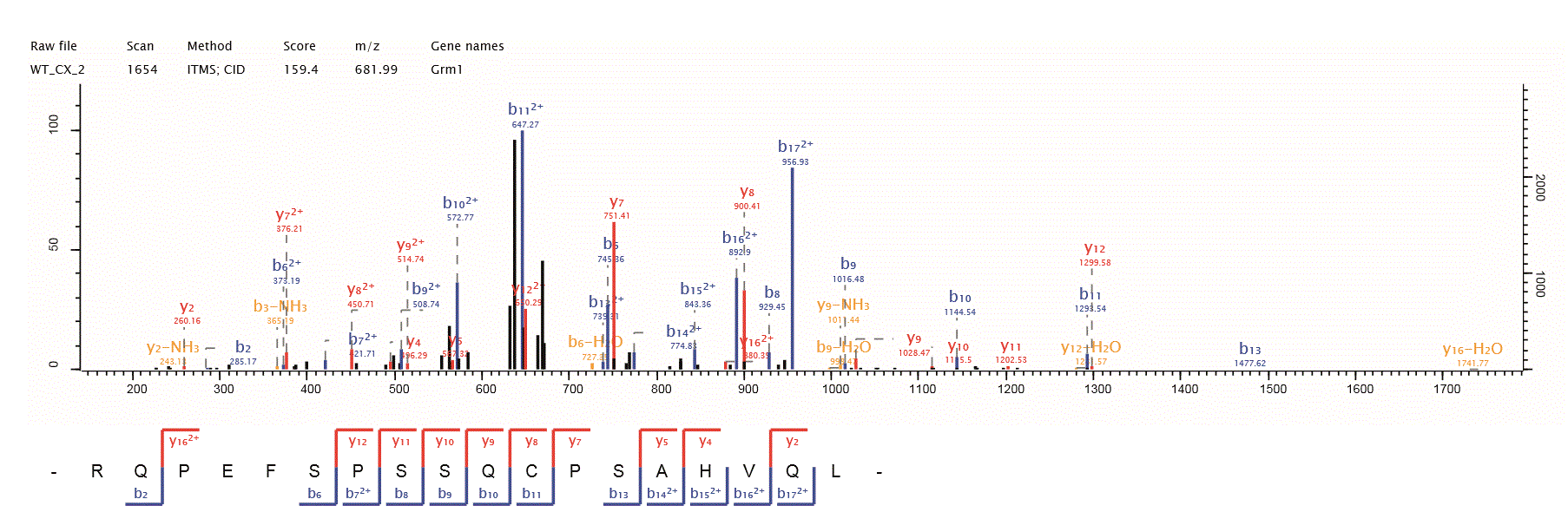

Supplement: Supplementary file 1 — Suppl. Figure S1 [file PMIC-16-2698-s001.GIF]
